# Role of prothrombin complex concentrate in the severe trauma patient

**DOI:** 10.3389/fmed.2022.988919

**Published:** 2022-11-02

**Authors:** Juan José Egea-Guerrero, Manuel Quintana-Diaz

**Affiliations:** ^1^Neurocritical Care Unit, Hospital Universitario Virgen del Rocío, Seville, Spain; ^2^Institute of Biomedicine (IBiS)/Consejo Superior de Investigaciones Científicas (CSIC) University of Seville, Seville, Spain; ^3^Intensive Care Unit, Hospital Universitario de La Paz, Madrid, Spain

**Keywords:** prothrombin, massive bleeding, coagulation, trauma-induced coagulopathy, bleeding management protocol

## Introduction

Traumatic hemorrhagic shock causes an estimated annual loss of more than 75 million years of life globally, and mostly affects young people (1). It is a time-dependent process, in which prehospital and emergency interventions must be sufficient to prevent the lethal triad of hypothermia, acidosis, and coagulopathy (2), and to avoid trauma-induced coagulopathy (TIC). Both are key determinants of trauma-associated mortality, so early control is essential to improve prognosis and reduce this outcome (3). During hemorrhagic shock, special attention needs to be paid to hyperfibrinolysis and deficiency of coagulation factors. The latter may be deficient for various reasons, such as loss due to hemorrhage, dilution due to vigorous fluid resuscitation, or the activation of pathophysiological mechanisms in TIC that mediate dysfunction (1, 4, 5).

From a therapeutic point of view, the administration of fresh frozen plasma (FFP) has conventionally been the only option to compensate TIC. However, this strategy is associated with a number of limitations such as fluid overload, variability in the quantity and quality of coagulation factors administered, as well as the need for compatibility with the patient's blood group and thawing prior to infusion. The use of FFP has also been associated with an increased risk of respiratory distress and multiple organ failure (6).

Prothrombin complex concentrate (PCC) could be considered an option within the emergent management of severe trauma patients, given its advantages such as lower infusion volume, immediate bioavailability, and compatibility with all blood groups. Additionally, a correct assistance of these patients requires a time-dependent management, thus, PCC can be administered immediately which makes it highly favorable. It is important to note that the composition of individual coagulation factors and the content of anticoagulants, especially heparin, as well as the indications vary among PCC from different manufacturers (7, 8). Therefore, their function and indications also differ.

## Use of prothrombin complex in the undercoagulated patient

In recent years, the management of undercoagulated patients has improved with the introduction of new direct-acting oral anticoagulants, such as thrombin and factor X inhibitors. Nonetheless, the incorporation of these drugs into the standard therapeutic arsenal of this population presents a challenge for urgent reversal of coagulation in the undercoagulated severe trauma patient (9). Although these direct-acting drugs have antidotes, not all are marketed in Spain, despite being authorized by the Spanish Agency for Medicines and Medical Devices (AEMPS). Accordingly, in these cases, the therapeutic alternatives involve waiting for the half-life of the drug, dialyzing the patient, if feasible, or administering high doses of PCC (25–50 IU/kg) together with tranexamic acid (9, 10). Studies have identified the reversal capacity of this strategy, reporting a good safety profile, and indeed the authors of one such paper recommend performing a comparison between PCC and andexanet alfa (antidote against thrombin inhibitors) to establish the best alternative in each case (7).

However, the approved indications for PCC are limited to antagonizing the effect of vitamin K-dependent coagulation factor inhibitors and treating congenital coagulation factor deficiency. The latest guidelines for severe polytrauma patients recommend the direct application of PCC in the emergent reversal of patients anticoagulated with vitamin K antagonists, based on the patient's weight and international normalized ratio (INR) (11). Furthermore, observational studies in traumatic brain injuries have found that PCC, as in spontaneous intracerebral hemorrhage, rapidly normalizes the INR, limiting the intracerebral injury and improving the prognosis of these patients (12).

## Application of PCC in the urgent management of the polytrauma patient

Although some experimental studies report rapid recovery of hemostasis with the use of PCC, its application in the urgent management of the polytrauma patient with TIC is underdeveloped (13). This may be due in part to concerns over the risk of using a drug that increases the availability of thrombin and, therefore, the patient's thromboembolic risk. However, evidence regarding the reversal of coagulation in anticoagulated patients shows that the risk—estimated to be around 1.8%—is relatively low (14).

It is important to note that the pharmacological management of TIC should not focus solely on the urgent infusion of coagulation factors in the form of PCC. The possibility of administering both fibrinogen and tranexamic acid must first be evaluated (3, 4, 10). Moreover, the accumulation of thrombin that cannot interact with fibrinogen will not result in the fibrin necessary to generate the clot; furthermore, if hyperfibrinolysis occurs, the TIC will not be reversed. In fact, the isolated use of PCC in trauma does not allow optimization of viscoelastic test parameters unless it is combined with the administration of fibrinogen (15). Specifically, the parameter that should guide our actions when deciding to administer PCC is extrinsic thromboelastometry [EXTEM CT (clotting time)], provided that fibrinogen values have been restored (10). In this regard, it should be noted that the use of PCC has been reported to reduce the use of blood products compared to FFP (16). However, in this study, the control group was a historical cohort in which the baseline blood fibrinogen concentration was not considered, possibly generating bias. Other studies suggest that one benefit of PCC is that fewer transfusions are required after its use; even so, the authors are cautious about the use of PCC as first-line therapy in the management of severe polytrauma patients (17).

## When should we use prothrombin complex to reverse TIC?

Various factors should be considered when deciding when to use PCC to reverse TIC: (i) Is this a massive bleed? (ii) Is there compromised space or organ-specific involvement (e.g., central nervous system, pharyngeal hematoma, intraocular hematoma, etc.)? (iii) Is there persistent major bleeding? (iv) Is urgent surgery or surgery with high bleeding risk needed? and (v) Are no other time-dependent options available (as happens in situations of war or catastrophes, etc.,)? Finally, the criterion of the healthcare team should carry a specific weight when determining the use of PCC. Given that early initiation of all medical-surgical interventions is essential for the successful management of TIC, we found studies that have evaluated the role of the normalization of hemostasis during the prehospital care of patients by emergency teams, some of which were successful and others somewhat contradictory (18, 19).

**Figure 1:**
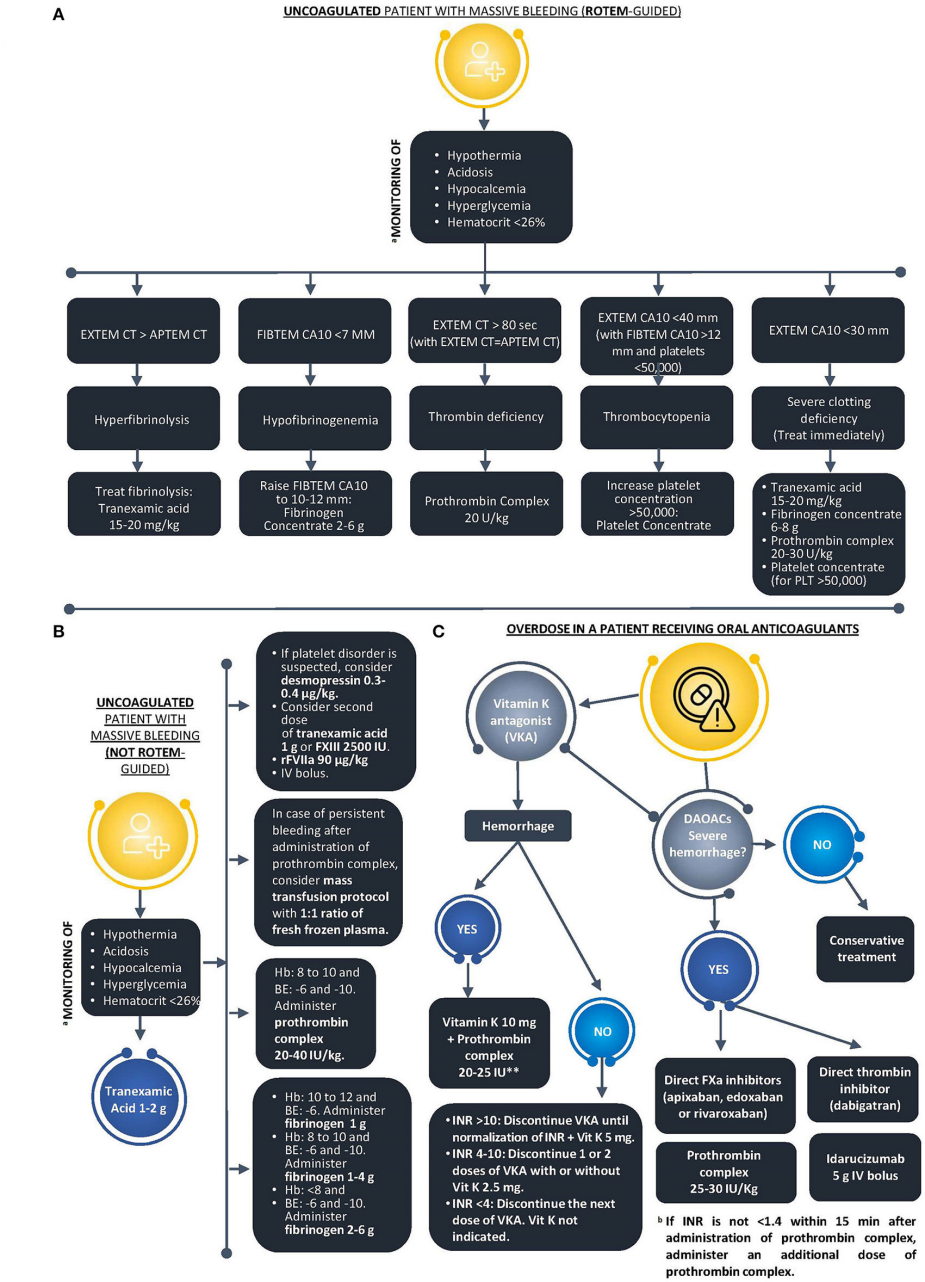
Coagulation support scheme for massive bleeding in two different scenarios: **(A)** Uncoagulated patient with massive bleeding (ROTEM-guided). **(B)** Uncoagulated patient with massive bleeding (not ROTEM-guided). **(C)** Overdose in a patient receiving oral anticoagulants. ^a^The presence of the lethal triad is not a prerequisite to activate the algorithm; however, we stress the need to look for those signs during the approach of severe polytrauma patients. ^b^Fixed doses 1000 UI. APTEM, aprotinin thromboelastometry; APTT, activated partial thromboplastin time; CT, clotting time; EXTEM, extrinsic thromboelastometry; FIBTEM, fibrinogen thromboelastometry; Hb, hemoglobin; HEPTEM, heparinase-modified thromboelastometry; INR, international normalized ratio; INTEM, intrinsic thromboelastometry; MCF, maximum clot firmness; ML, maximum lysis; PCC, prothrombin complex concentrate; PCF, platelet contractile force; PT, prothrombin time; ROTEM, rotational thromboelastometry.

It is important to note that in order to have objective parameters on which to base the management of TIC in emergencies, the use of PCC must always be guided, either by conventional tests, such as INR, or by the support of viscoelastic tests (in case of EXTEM CT >80 s) (10, 16). In this regard, the algorithm presented herein (Figure 1) may be very useful for decision-making in these patients. The logistics and specific circumstances of each emergency unit must be taken into consideration, so that this algorithm can be adapted to the local protocol.

## Conclusion

Limited therapeutic strategies are available today in the management of TIC, and those that are, are based on the correction of coagulopathy triggers and associated processes, and the replacement of coagulation factors. PCC is a useful therapeutic option with a proven cost-effectiveness balance in different emergency departments in Spain that should be analyzed in greater detail in the near future (10). However, more data from clinical trials are needed if we are to offer our severe trauma patients safe and personalized interventions. We must not forget that while increasing thrombin production is a fundamental process in the management of patients in traumatic hemorrhagic shock, fibrinolysis must be simultaneously controlled and platelet function and clot formation must be monitored. Otherwise, management will not be well balanced and the results may not be as expected, impacting on TIC and the outcome of the severe trauma patient.

## Author contributions

JE-G and MQ-D made a substantial contribution to the concept of the article. JE-G drafted the preliminary version of the article. Both authors read and approved the final version of the article.

## Conflict of interest

The authors declare that the research was conducted in the absence of any commercial or financial relationships that could be construed as a potential conflict of interest.

## Publisher's note

All claims expressed in this article are solely those of the authors and do not necessarily represent those of their affiliated organizations, or those of the publisher, the editors and the reviewers. Any product that may be evaluated in this article, or claim that may be made by its manufacturer, is not guaranteed or endorsed by the publisher.
